# Long-Term Treatment With Letrozole in a Boy With Familial Male-Limited Precocious Puberty

**DOI:** 10.3389/fendo.2022.906852

**Published:** 2022-07-14

**Authors:** Xin Yuan, Ruimin Chen, Ying Zhang, Xiaohong Yang, Xiangquan Lin

**Affiliations:** Department of Endocrinology, Genetics and Metabolism, Fuzhou Children’s Hospital of Fujian Medical University, Fuzhou, China

**Keywords:** familial male-limited precocious puberty, aromatase inhibitor, control of puberty, gene diagnostics, follow-up

## Abstract

**Background:**

The long-term follow-up in children with familial male-limited precocious puberty (FMPP) who were treated with letrozole, triptorelin, and spironolactone is limited, especially considering the efficiency and safety.

**Objective:**

We describe the clinical characteristics and long-term treatment with letrozole on adult height of a boy diagnosed with FMPP, confirmed by analysis of the LHCGR gene.

**Methods:**

Physical examinations, bone age (BA), testosterone, and gonadotropin levels were measured as well as gene sequencing of the proband and parents.

**Results:**

The boy was referred to the hospital at 3.1 years of age due to peripheral precocious puberty. His height was 116.8cm (+5.1SD) and BA was 9 years. Genetic analysis revealed a patrilineal c.1703C>T.(p.Ala568Val) mutation of the LHCGR gene. After treating with letrozole for 1.6 years, the height according to BA went from -3.52SD to -2.82SD. Triptorelin was added at age 4.7 years based on both the evidence of central puberty and his growth velocity according to BA. During the 6.9 years of treatment, he had a height gain of 51.9cm, and BA increased 5.2 years. At age 10, his present height is 168.7cm (0.05SD) and BA is 14.7 years. No adverse effects of treatment were encountered.

**Conclusion:**

A patrilineal mutation of the LHCGR gene has been identified in a boy with FMPP. His height is 168.7cm (-0.05SD) which is approaching his adult height after long-term treatment with letrozole, triptorelin, and spironolactone.

## Introduction

Familial male-limited precocious puberty (FMPP), also known as testotoxicosis, is a rare form of gonadotropin-independent precocious puberty. It is caused by heterozygous constitutively activating mutations of the luteinizing hormone/choriogonadotropin receptor (LHCGR) gene. The mutation can occur *de novo* or be inherited in an autosomal dominant fashion. The phenotype is limited to males. The age of onset is usually 2-4 years, and it is characterized by rapid virilization including an enlarged penis, minimal testicular enlargement, pubic hair development, accelerated linear growth, and skeletal maturation, which begets a reduced final adult height (1).

In order to reduce the synthesis and action of androgens, several agents such as medroxyprogesterone, ketoconazole, and cyproterone have been adopted for treatment (2). Aromatase inhibitors are also included in treatment regimens to curtail the bone age (BA) advancement, which can result in encouraging short-term results (3, 4). However, given the rarity of FMPP, there is a paucity of long-term information with these agents, especially with letrozole.

Thus, the aim of this study is to describe the clinical characteristics and long-term follow-up of a Chinese boy with FMPP. He is the first Chinese Han boy treated with a combination of letrozole, triptorelin (gonadotropin-releasing hormone analog, GnRHa), and spironolactone.

## Patient and Methods

### Patient

#### Clinical and Anthropometric Evaluation

The patient was assessed at diagnosis and scheduled for outpatient visits every 3-6 months during treatment. Characteristics from clinical examinations and medical records encompass: anthropometric data including weight (in kg) and height (in cm), Tanner pubertal staging, and medications. Anthropometric data are depicted in absolute values and converted into Z scores (5). Target height was calculated as ((paternal height + maternal height)/2 + 6.5) cm.

### Methods

#### Informed Consent

Parents provided written consent for hormonal tests, molecular study, and permission to publish the case.

#### Serum Laboratory Tests

Fasting blood glucose, blood lipids, and liver enzymes were assayed by Abbott and specific reagents (Abbott, Abbott Park IL, USA). Total testosterone, baseline and gonadotropin-releasing hormone (GnRH)-stimulated follicle-stimulating hormone (FSH), luteinizing hormone (LH), 17-alpha-hydroxyprogesterone (17-OHP), androstenedione (AND), dehydroepiandrosterone sulfate (DHEAS), free thyroxin (FT4), thyroid-stimulating hormone (TSH), and cortisol were analyzed by chemiluminescent immunoassay method (Siemens, Erlangen, Germany SIEMENS IMMULITE 2000 and specific reagents, Germany). Once GnRHa was initiated, GnRH stimulation testing was performed every 12 months to ensure suppression of secondary central puberty (suppressed basal and peak LH and FSH levels). BA was assessed by Greulich−Pyle method at diagnosis and each subsequent visit.

#### Molecular Screening for the Mutation in the LHCGR Gene

After informed consent, peripheral venous blood from the patient and his parents was collected, and genomic DNA from peripheral blood leucocytes was isolated using the QIAamp Blood DNA Mini Kit (QIAGEN, Hilden, Germany) following the manufacturer’s instructions. All 11 exons and 10 introns of LHCGR were amplified using AmpliTaq DNA polymerase (Applied Biosystems, Foster City CA, USA) for Polymerase Chain Reaction (PCR) amplification from 100 ng of genomic DNA, and primers were designed by Primer Premier 5.0 Software. The PCR products were directly sequenced using an ABI 3130 x 1 Genetic Analyzer (Applied Biosystems, Foster City CA, USA). Sequencing results were compared to the reference data available at the NCBI RefSeq database NC_000002.12 (GRCh38) for LHCGR gene.

## Results

### Clinical and Hormonal Features of the Patient With FMPP

The boy was referred to the Endocrinology Outpatient Clinics of Fuzhou Children’s Hospital of Fujian for evaluation of precocious puberty. He presented with an enlarged penis from the age of 3–4 months old and developed pubic hair at the age of 2.6 years in concert with accelerated growth. He was born spontaneously to healthy and unrelated parents of Chinese origin, and his neonatal course was unremarkable. Father is 165 cm (-1.26 SD) tall and mother is 152 cm (-1.59 SD). Target height is 165 cm (-1.26 SD). No similar medical history is present in other family members. At age of 3.1 years, a clinical exam revealed an 8.5 cm × 2.5 cm stretched penis and enlarged testes (8 mL bilateral, compatible with Tanner stage 2), pubic hair stage 2, height of 116.8 cm (+5.1 SD), and BA of 9.5 years. Gonadotropin levels were normal and serum total testosterone was disproportionally elevated, consistent with gonadotropin-independent precocious puberty (**Table 1**). Adrenal and thyroid serum profiles were normal. MRI of the hypothalamic-pituitary region was normal.

**Table 1:** Hormonal features of the patient with testotoxicosis at diagnosis.

	Detection value	Normal range
Basic LH (IU/L)	0.25	
Basic FSH (IU/L)	0.37	
Peak LH (IU/L)	2.66	
Peak FSH (IU/L)	1.03	
total testosterone (ng/dl)	231	<20
Androstenedione (μg/L)	<0.3	0.7-3.6
dehydroepiandrosterone sulfate (μg/dL)	<15	80-560
sex hormone binding globulin	80.1	13-71
Progesterone (μg/L)	1.18	0-1.4
17-alpha-hydroxyprogesterone (nmol/L)	2.0	0-30
human chorionic gonadotrophin (U/L)	0.91	0.4-5.0
Thyrotropin (U/L)	0.91	0.4-5.0
Free triiodothyronine (ng/L)	3.10	1.5-4.1
Free thyroxine (pmol/L)	21.60	8.3-29.6
Adrenergic hormone releasing hormone (ng/L)	30.2	0-46
Cortisol (nmol/L)	241	138-690
Growth hormone (μg/L)	1.95	
Insulin - like growth factor-1 (μg/L)	363	49-297

LH: luteinizing hormone; FSH: follicle-stimulating hormone; Peak LH value and peak FSH value were measured after exogenous intravenous administration of luteinizing−hormone releasing hormone (2.5ug/kg).

The boy’s father is 35 years old, basal LH 0.56 IU/L (normal range: 0.8-7.6 IU/L), and FSH 1.82 IU/L (normal range: 0.7-11.1 IU/L), serum total testosterone level was 5000 ng/dl (normal range: 2620-15930 ng/dl), DHEAS 712 μg/l (normal range: 1200-5200 μg/l), and androstenedione 2.18 μg/l (normal range: 0.7-3.6 μg/l).

**Figure 1:**
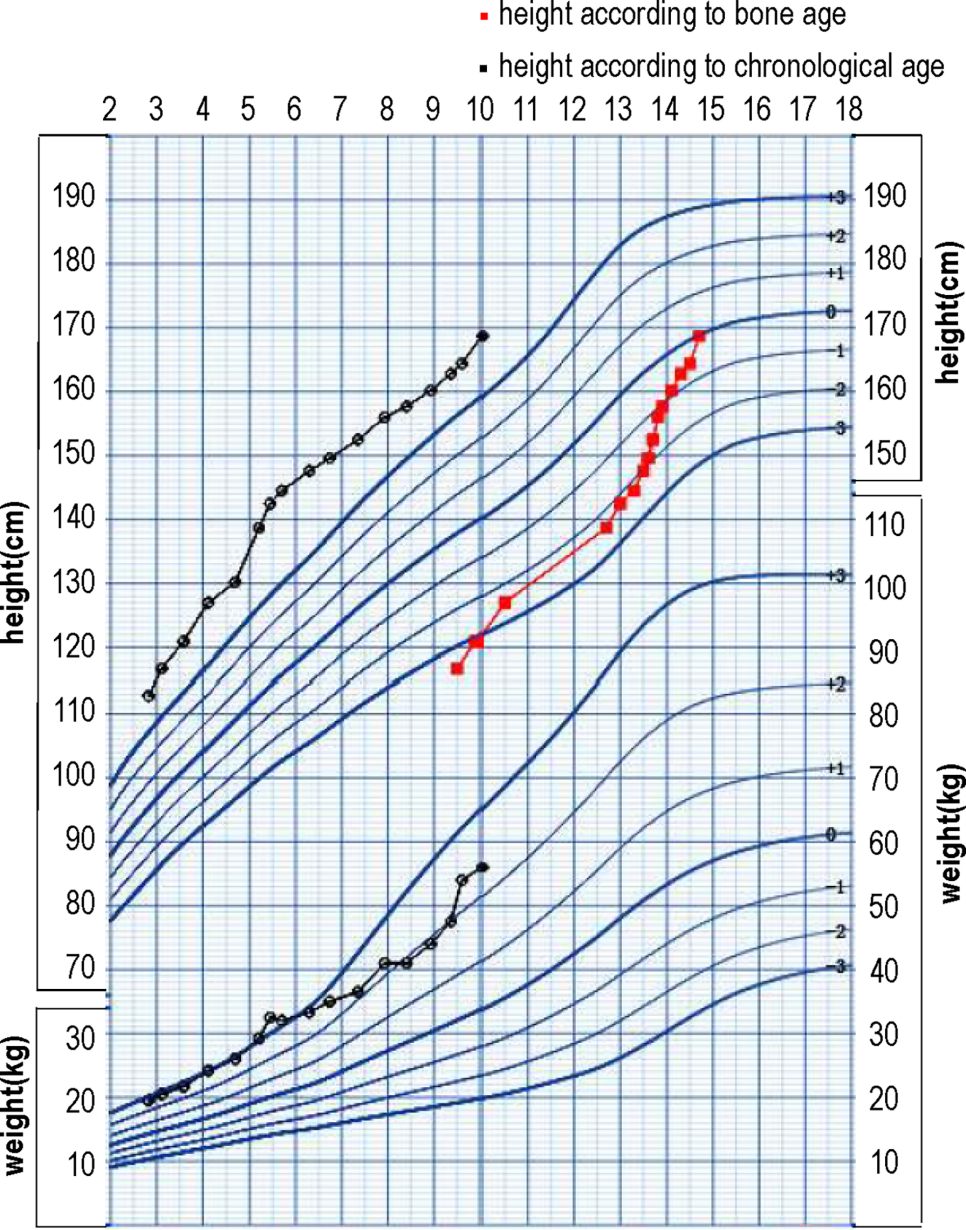
The growth chart of the patient with height and weight, growth velocity, along with the height according to bone age.

At 3.1 years old, the boy was treated at diagnosis with letrozole at a dose of 1.25 mg/d (0.06 mg/kg/d). At his 2-month follow-up visit, his parents reported that the frequency of penis erection decreased from several times per day to several times per month. The boy was diagnosed with secondary central puberty at 3.8 years based on both clinical evidence (increased volume of testicles) and a GnRH stimulation test (peak LH 4.21 IU/L, peak FSH 1.76 IU/L). The growth velocity according to BA during the first 0.7 years of the treatment was 11cm/yr. However, the growth velocity according to BA for the next 0.9 years decreased to 4.4 cm/yr. Considering the decreased growth velocity and unsatisfactory height according to BA (-2.82 SD), triptorelin was added at 4.7 years of age. During the 3.9 year treatment with letrozole and triptorelin, the BA increased 2.3 years and growth velocity according to BA increased to 12.5cm/yr. At 5.5 years old, the dose of letrozole was added to 2.5 mg/d (0.08 mg/kg/d). Because his elevated serum testosterone persisted (306 ng/dl), spironolactone was added to a dose of 20 mg tid. In sum, during the 6.9 years of treatment, he gained 51.9 cm in height and his BA increased by 5.2 years. No drug-related severe adverse events occurred during treatment. Anthropometric data and hormonal profile of the boy are summarized in **Table 2**. The height and weight, growth velocity, along with height according to bone age are shown in **Figure 1**.

**Table 2:** follow-up of the patient with testotoxicosis.

drug	CA (year)	Treatment time (year)	Height (cm)	HtSDS	BA (year)	BA htSDS	total testosterone (ng/dl)
Letrozole	3.1	0	116.8	4.76	9.5	-3.52	363
	3.8	0.7	122.3	4.8	10	-2.89	227
	4.1	1	127	5.26	ND	ND	ND
Letrozole + Triptorelin	4.7	1.6	130.2	4.82	11.8	-2.82	101
Letrozole + Triptorelin+ Spironolactone	5.5	2.4	142.5	6.18	13	-2.21	209
	5.8	2.7	144.5	6.03	13.3	-2.25	161
	6.8	3.7	149.6	5.32	13.6	-1.86	40
	7.9	4.8	156	4.71	13.8	-1.21	94
Letrozole+ Spironolactone	8.6	5.5	159	4.60	14.1	-1.05	165.3
	8.9	5.8	160.2	4.37	14.2	-0.96	535
	9.4	6.3	162.8	4.17	14.3	-0.66	1339
withdrawal	10	6.9	168.7	4.60	14.7	-0.02	1431

CA, Chronological age; BA, bone age; ND, not done.

### Mutation Detection

Molecular analysis revealed a nucleotide transition c.1703C>T, leading to a heterozygous amino acid substitution at codon 568 (p.Ala568Val), a mutation within the hot spot of the LHCGR gene. His father carried the same heterozygous mutation, whereas his mother had no such mutation.

## Discussion

The human LHCGR is a member of the G protein-coupled receptor family and consists of 11 exons and 10 introns with seven transmembrane helices, located on the short arm of chromosome 2 (2p21) (6). LHCGR gene mutations have been described in several populations such as Brazilian (6), American (7), and Japanese (8). A total of 16 mutations had been reported (limited missense variants). Activating mutations identified in boys with FMPP are mostly located in exon 11, resulting in LHCGR highly expressed in testicular Leydig cells, with resultant excessive testosterone production (9). The restricted number of LHCGR gene mutations found in familial and sporadic cases strongly infer that only mutation in specific domains of the receptor can autonomously activate cAMP cell signaling (6). Furthermore, female patients who carry LHCGR-activating mutations do not present with clinical or hormonal abnormalities, indicating the lack of phenotypic expression of these mutations (10).

FMPP is exceptionally unusual in the Chinese population, considering that only two patients have been genetically confirmed (11, 12). We described a boy presenting with an early and severe gonadotropin-independent precocious puberty carrying a patrilineal inherited p.Ala568Val mutation in the LHCGR which, therefore, has not been reported in the Chinese population. The clinical and hormonal profile of the boy was similar to those of patients with the mutation p.Ala568Val previously described in other populations (10, 13–15). This mutation substitutes alanine 568 with valine at the carboxyterminus of the third cytosolic loop of the LH receptor. The unoccupied mutant receptors confer constitutive activation of adenyl cyclase activity when expressed in COS-7 cells, resulting in four-fold higher cAMP concentrations over baseline compared with cells expressing an equivalent number of wild-type receptors. The affinity of the mutant receptors to 125I-labeled human LH was not altered compared with the wild type. Alanine residue is crucial for signal transduction and a potential site for upregulatory/oncogenic mutations in G-protein coupled receptors 13).

The father of our patient carried the same mutation in the LHCGR gene, however, he showed none of the salient clinical manifestations. However, he did have a lower LH level compared with his peers. In this disorder, genotype does not always correlate with phenotype (16). Jeha GS et al. (7) reported three family members with a D564G mutation of the LHCGR gene. All three males had precocious puberty with elevated testosterone levels. Yet the phenotypic expression varied from severe precocity unresponsive to therapy with a compromise of the predicted final height in some members, to attainment of tall final stature in other untreated members. In sum, the possibility that the effects of the mutant LHCGR on phenotypic expression of FMPP, such as adult height, are modified by other factors.

Considering that the time available for growth is limited, early treatment of FMPP is imperative. Selective suppression of estrogen production or action can increase height potential by delaying epiphyseal fusion (1). However, there is no consensus as to treatment modality and conflicting regimens have been proposed. Treatment of FMPP has traditionally targeted steroidogenesis by ketoconazole and antiandrogens, such as cyproterone acetate, and spironolactone. Aromatase inhibitors have been championed to mitigate the effect of increased estrogen on skeletal maturation (1). Ketoconazole inhibits adrenal and testicular androgen biosynthesis, but severe liver damage has been reported in a child receiving high-dose therapy (1,200 mg/day) (17). Almeida MQ et al. (18) reported a multicentric, retrospective, long-term treatment study in which 10 patients were evaluated. Cyproterone acetate was administered to five patients and ketoconazole to five others. Nonetheless, both agents had limited effectiveness on final height. Furthermore, considering the endocrine dysregulation and hepatotoxicity, market withdrawal of ketoconazole has taken place in some countries and, in others, product guidelines and labels are enforced (19).

The third-generation aromatase inhibitors, such as the nonsteroidal anastrozole and letrozole, can be taken orally once daily without significant side effects. These compounds reduce serum estrogen by the inhibition of P450 aromatase. Several reports have found that aromatase inhibitors normalize growth velocity and the rate of bone maturation with an improved PAH (20, 21). In other pediatric conditions associated with short stature, these agents have been presented, such as constitutional delay of growth and adolescence (22), growth hormone deficiency (23), and idiopathic short stature (24). Aromatase inhibitors and anti-androgen agents were effective in reducing virilization and in decreasing testosterone synthesis without side effects (25). The BATT study (Bicalutamide and Anastrozole Treatment of testotoxicosis), a multicenter, one-year, industry-sponsored study of 14 boys with FMPP using the newer androgen receptor antagonist bicalutamide, along with the third-generation AIs anastrozole, found that these agents were effective in slowing growth velocity and bone age advancement with once-daily dosing (26). Lenz et al. (4) reported two boys treated with anastrozole and bicalutamide, which was well-tolerated and effective in preventing progression of virilization and BA with no decrease in linear growth. However, all these studies were short-term.

Anastrozole is rapidly absorbed (maximum after 1 h) and slowly eliminated (terminal half-life of 46.8 h) after oral dosing. Letrozole is also rapidly absorbed but has a longer half-life (2–4 days) and suppresses aromatase activity more so than anastrozole, as evidenced by the higher plasma testosterone and gonadotropin levels (27). Anastrozole is widely used in the treatment of FMPP (8, 26, 28). Only one study reported using letrozole to treat FMPP for 4.5 years, which increased the PAH (4) . However, long-term treatment of FMPP with letrozole until near-final height has not been reported.

In our study, when the child with FMPP was 3.1 years old, the GnRH stimulation test revealed a peak LH of 2.66 IU/L and FSH of 1.03 IU/L, inconsistent with the diagnostic criteria for central precocious puberty. As a result, the boy was treated with letrozole alone. After treatment with letrozole for 2 months, his parents reported that the frequency of penile erection decreased from several times per day to several times per month. A reduction in testosterone level was also observed at 3.8 years old (treated with letrozole for 0.7 years). Nabhan ZM (29) reported spontaneous remission in a boy with FMPP, characterized by prepubertal testosterone levels (10 to 28 ng/dL). However, the growth rate of our patient was not decreased during the treatment. After letrozole for 1.6 years, at 4.7 years old, the GnRH stimulation test revealed a peak LH of 4.21 IU/L, the LH/FSH ratio was increased, and his BA advancement was inadequately suppressed. As a result, triptorelin was added to inhibit the hypothalamic-pituitary-gonadal axis.

The patient in this study was treated from early childhood with letrozole for 1.6 years, combined with GnRHa for 3.9 years after premature secondary gonadotropin activation, and an antiandrogen was added because of the sustained high level of testosterone. At present, BA of the patient is 14.7 years old and height is 168.7 cm -0.05 SD). Recently, Leschek EW et al. (28) reported 28 boys with FMPP treated with antiandrogen combined with AIs, and GnRHa was added based on evidence of central puberty. Adult height (mean ± SD) for all treated subjects was 173.6 ± 6.8 cm (−0.4 ± 1.0 SD relative to adult US males), which was a superior response compared to our boy. Several factors may explain the differences: 1) BA of the patient in our study was advanced 6.4 years at treatment onset, far more worrisome than the 4.8 years advanced in the just-cited report. 2) Anastrozole rather than letrozole was used. Neely EK et al. investigated the short- and long-term hormonal and auxologic differences in short pubertal boys treated with letrozole or anastrozole: the former was more potent in hormonal manipulation than anastrozole. First-year growth velocities were comparable, but improvement in PAH was greater in the anastrozole group. It remains to be seen if positive PAH trends will translate to an increase in final height in either group (30).

With more potent AIs and antiandrogens now available, it may be possible to block the effects of central puberty with a combined AI and antiandrogen regimen, thereby avoiding the high cost and burden of GnRHa treatment (23). In Leschek EW et al.’s report (26), the study design does not allow the determination of differences in effectiveness between initial and subsequent choices of AI or GnRHa. Thus, the results should be viewed as a proof of concept for combined antiandrogen and AI treatment to normalize growth rate, bone maturation, and pubertal progression to achieve a near-normal adult height.

## Conclusion

In conclusion, a Chinese boy with classic clinical and hormonal characteristics of FMPP is described. Long-term treatment with letrozole and triptorelin has led to satisfactory results despite the fact that his BA was advanced 6.4 years prior to treatment. Of note, the 6.9 years of treatment was effective in attenuating the worrisome BA advancement, yet did not impair his liner growth. No side effect was observed during the treatment.

## Data Availability Statement

The original contributions presented in the study are included in the article/supplementary material. Further inquiries can be directed to the corresponding author.

## Ethics Statement

The studies involving human participants were reviewed and approved by the Ethics Committee of Fuzhou Children’s Hospital of Fujian Medical University. Written informed consent to participate in this study was provided by the participant’s legal guardian/next of kin.

## Author Contributions

XY drafted the initial manuscript. RCM diagnosed the patient and developed treatment plan, and reviewed and revised the manuscript. YZ and XHY collected data. XLL did the laboratory testing. All authors contributed to the article and approved the submitted version.

## Funding

This study was sponsored by Key Clinical Specialty Discipline Construction Program of Fuzhou, Fujian, P.R.C (201610191).

## Conflict of Interest

The authors declare that the research was conducted in the absence of any commercial or financial relationships that could be construed as a potential conflict of interest.

## Publisher’s Note

All claims expressed in this article are solely those of the authors and do not necessarily represent those of their affiliated organizations, or those of the publisher, the editors and the reviewers. Any product that may be evaluated in this article, or claim that may be made by its manufacturer, is not guaranteed or endorsed by the publisher.
